# Functional identification of an opsin kinase underlying inactivation of the pineal bistable opsin parapinopsin in zebrafish

**DOI:** 10.1186/s40851-021-00171-1

**Published:** 2021-02-12

**Authors:** Baoguo Shen, Seiji Wada, Haruka Nishioka, Takashi Nagata, Emi Kawano-Yamashita, Mitsumasa Koyanagi, Akihisa Terakita

**Affiliations:** 1 Department of Biology and Geosciences, Graduate School of Science, Osaka City University, Osaka, 558-8585 Japan; 2 The OCU Advanced Research Institute for Natural Science and Technology, Osaka City University, Osaka, 558-8585 Japan; 3 Department of Chemistry, Biology, and Environmental Science, Faculty of Science, Nara Women’s University, Kitauoyanishi-machi, Nara, 630-8506 Japan

**Keywords:** Nonvisual photoreception, Pineal organs, Bistable opsin, G-protein-coupled receptor kinase, Opsin inactivation

## Abstract

In the pineal organ of zebrafish larvae, the bistable opsin parapinopsin alone generates color opponency between UV and visible light. Our previous study suggested that dark inactivation of the parapinopsin photoproduct, which activates G-proteins, is important for the regulation of the amount of the photoproduct. In turn, the photoproduct is responsible for visible light sensitivity in color opponency. Here, we found that an opsin kinase or a G-protein-coupled receptor kinase (GRK) is involved in inactivation of the active photoproduct of parapinopsin in the pineal photoreceptor cells of zebrafish larvae. We investigated inactivation of the photoproduct in the parapinopsin cells of various knockdown larvae by measuring the light responses of the cells using calcium imaging. We found that GRK7a knockdown slowed recovery of the response of parapinopsin photoreceptor cells, whereas GRK1b knockdown or GRK7b knockdown did not have a remarkable effect, suggesting that GRK7a, a cone-type GRK, is mainly responsible for inactivation of the parapinopsin photoproduct in zebrafish larvae. We also observed a similar knockdown effect on the response of the parapinopsin photoreceptor cells of mutant larvae expressing the opsin SWS1, a UV-sensitive cone opsin, instead of parapinopsin, suggesting that the parapinopsin photoproduct was inactivated in a way similar to that described for cone opsins. We confirmed the immunohistochemical distribution of GRK7a in parapinopsin photoreceptor cells by comparing the immunoreactivity to GRK7 in GRK7a-knockdown and control larvae. These findings suggest that in pineal photoreceptor cells, the cone opsin kinase GRK7a contributes greatly to the inactivation of parapinopsin, which underlies pineal color opponency.

**Supplementary Information:**

The online version contains supplementary material available at 10.1186/s40851-021-00171-1.

## Background

The pineal and related organs of most nonmammalian vertebrates have direct light sensitivity [1–7]. Interestingly, the organs in the lamprey, teleost, frog, and lizard have the ability to discriminate between the wavelengths of UV and visible light, in addition to their detection of irradiance [1–3, 8].

We previously showed that parapinopsin, a pineal opsin first identified in the catfish pineal and parapineal organs [9], seemed to underlie the UV sensitivity of pineal wavelength discrimination in a lamprey [10–12], some teleosts [13, 14], and an iguana [15]. Parapinopsin is a UV-sensitive opsin that activates the G-protein transducin in a light-dependent manner, which leads to the hyperpolarization of pineal photoreceptor cells [10, 14, 16]. Although parapinopsin has an amino acid sequence similar to those of vertebrate visual opsins, such as rhodopsins and cone visual opsins [9, 10, 14, 16], and drives a similar biochemical cascade, it exhibits a different molecular property from that of visual opsins. The photoproduct of parapinopsin is thermally stable and reverts to the original dark state by subsequent light absorption, showing a bistable nature, whereas the photoproducts, i.e., meta II states, of rhodopsins and cone opsins are unstable and release chromophores [10, 14, 16].

Recently, we suggested that the bistable nature of parapinopsin contributes to physiology. In zebrafish pineal photoreceptor cells, under white light, parapinopsin 1 (PP1) [13, 17] alone generated color opponency between UV and visible light [14]. Opposite responses (hyperpolarization and depolarization to UV- and visible light-rich white lights, respectively) were generated by a wavelength-dependent shift in the photoequilibrium of the dark-inactive and light-activated states based on this bistable property [14]; i.e., the amount of the active photoproduct, which activates G-protein, determines the membrane potential level of the photoreceptor cells. In this context, we suggested that the rate of the photoproduct inactivation in the dark significantly affects the amount of the active photoproduct and, consequently, color opponency in PP1-expressing cells (PP1 cells) [14]. Therefore, PP1 photoproduct inactivation in the dark is thought to be important for understanding the mechanism of color opponency under natural white light.

Several lines of evidence show that in visual cells, i.e., rods and cones, the dark inactivation of light-activated rhodopsin and cone opsins involves two key proteins, G-protein-coupled receptor kinase (GRK) and arrestin (or S-antigen) [18–28]. In rods, arrestin binding requires the phosphorylation of light-activated rhodopsin by GRK. In cones, the phosphorylation of activated cone opsins by GRK is an additional important step for the termination of light responses.

In zebrafish, three types of GRKs have been functionally identified as opsin kinases in visual cells. GRK1a is involved in inactivation of the rhodopsin photoproduct, meta II [29], and GRK1b and GRK7a are involved in cone opsin inactivation in cone cells [30–32]. The pineal organ in adult zebrafish was reported to express three types of GRKs: the cone-related GRKs GRK1b and GRK7a, as well as GRK7b, a paralog of GRK7a [29]. We attempted to perform double in situ hybridization for each of these GRKs and PP1 in the zebrafish pineal organs but did not obtain sufficiently clear signals for GRK mRNAs to indicate the colocalization of a GRK with PP1. Here, we functionally identified a GRK involved in the dark inactivation of light-activated PP1 in zebrafish larvae via a combination of GRK knockdown using morpholino oligonucleotides and calcium imaging of PP1 cells after light activation. We also compared the contribution of the identified kinase to dark inactivation of the PP1 photoproduct and a cone opsin photoproduct with a zebrafish mutant in which UV-sensitive cone opsin was expressed instead of parapinopsin to test whether the kinase makes a special contribution to dark inactivation of the PP1 photoproduct.

## Results and discussion

**Fig. 1:**
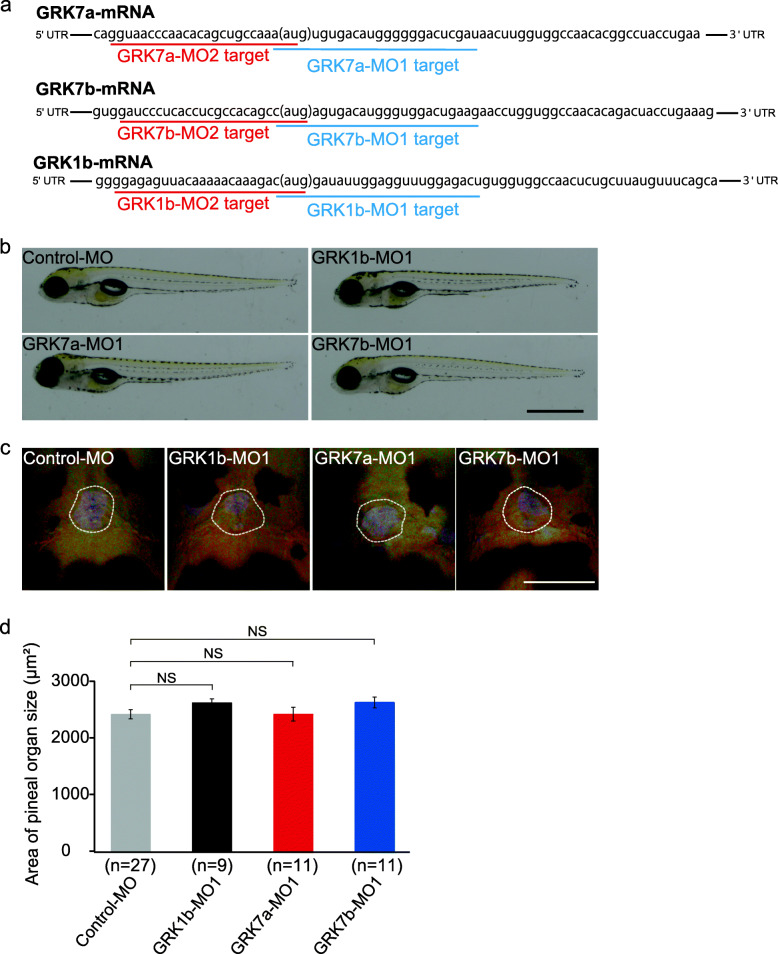
Morpholino oligonucleotide (MO)-mediated knockdown of different GRKs in zebrafish larvae. **a** Nucleotide sequences of different GRK mRNAs in zebrafish. Blue and red underlining indicates GRK-MO1s- and GRK-MO2s-targeted sequences, respectively. The start codons are indicated by brackets. **b** Images showing whole bodies of 5 dpf larvae injected with control, GRK1b, GRK7a or GRK7b MO1s. Scale bar represents 1 mm. **c** Dorsal view of the pineal organs of 5 dpf larvae injected with control or one of the GRK MO1s. Landmarks indicate the pineal organs. Scale bar represents 100 μm. **d** Quantitative analyses of pineal size (**c**) in larvae injected with control MO or one of the GRK MO1s. The error bars indicate the standard error. Significance scores represent *P* values determined with the Wilcoxon rank sum test (NS, nonsignificant, *P* > 0.05)

**Fig. 2:**
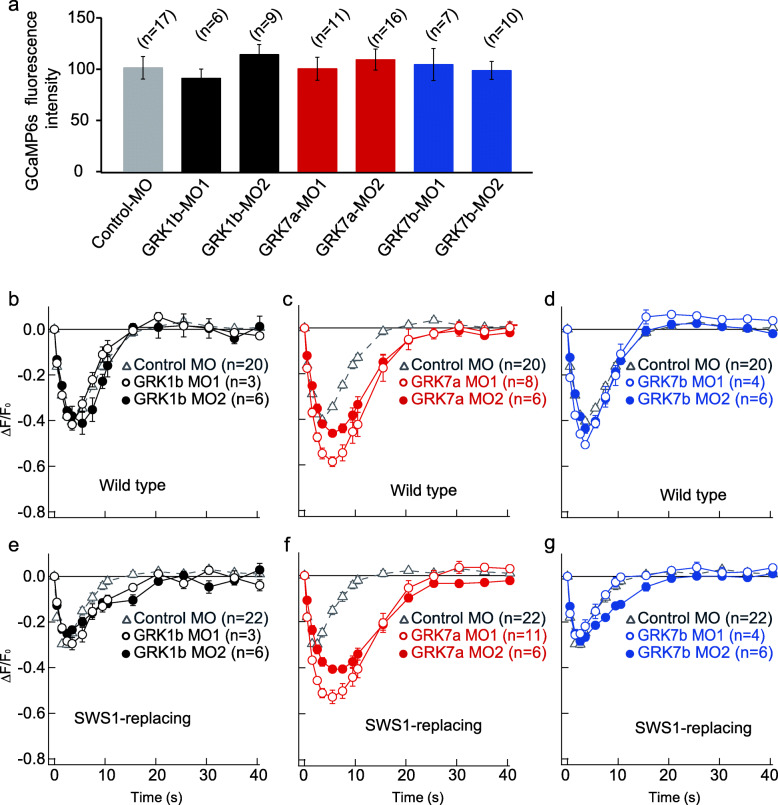
The light responses of PP1 cells in zebrafish larvae were affected by GRK knockdown. **a** Quantification of GCaMP6s fluorescence intensity in larvae injected with control MO or one of the GRK MOs. The error bars indicate the standard error. **b**-**g** Calcium changes upon 405 nm laser flashes showing the effects of GRK knockdown on light responses in the PP1 cells of wild-type larvae (**b**-**d**) and SWS1-replacing larvae (**e**-**g**). **b** and **e** GRK1b MO-knockdown larvae. GRK1b-MO1, black open circle; GRK1b-MO2, black closed circle. **c** and **f** GRK7a MO-knockdown larvae. GRK7a-MO1, red open circle; GRK7a MO2-knockdown larvae, red closed circle. **d** and **g** GRK7b MO-knockdown larvae. GRK7b-MO1, blue open circle; GRK7b-MO2, blue closed circle. Gray open triangles in b-g indicate control MO-injected larvae. The number of animals tested (n) is indicated in parentheses after the MO names in each panel. The error bars indicate the standard error. The duration of each stimulus was ~ 450 ms

Previous studies showed that GRK1b, GRK7a, and GRK7b are distributed in the pineal organs in zebrafish [29, 30]. We confirmed the expression of these GRKs in the pineal organ of zebrafish via in situ hybridization (Fig. S1). However, double-fluorescence in situ hybridization did not reveal the coexpression of GRK(s) with PP1 in the zebrafish pineal organs (Fig. S2), probably because the mRNA levels of GRKs in PP1 cells were insufficient for this method. Therefore, we investigated which of the three GRKs is involved in the inactivation of light-activated PP1 via a combination of calcium imaging of PP1 cells and GRK1b, GRK7a, or GRK7b knockdown with antisense morpholino oligonucleotides (MOs). We designed two kinds of MOs targeting each GRK mRNA (Fig. 1a) and confirmed that injection of each MO did not cause an abnormality in the whole-body morphology or size of the pineal organ (Fig. 1b-d and Fig. S3). Additionally, the GCaMP6s expression levels in the PP1 cells of MO-injected transgenic fish were similar to those of the control fish (Fig. 2a). We analyzed recovery of the calcium level in PP1 cells in the dark after a 405 nm light flash by measuring the difference in fluorescence intensity before (T = 0 s) and at a specific time point after (T = 0.5, 1, 2, 3, 5, 7, 9, 10, 15, 20, 25, 30, 35, or 40 s) stimulation with different MO-injected zebrafish larvae (5 dpf). Notably, by calcium imaging, we acquired only one image each before and after light stimulus and repeated the two image acquisitions to obtain a response profile to minimize opsin activation by the two-photon excitation light corresponding to blue light in the dark recovery process. We found that knockdown of GRK7a using the two kinds of MOs resulted in different response profiles, i.e., a larger amplitude at the peak, a longer duration of the peak, and a remarkably slower recovery of the calcium level in the PP1 cells compared with those from GRK1b-knockdown, GRK7b-knockdown, and control larvae (Fig. 2b-d, Fig. S4a, b), indicating that GRK7a plays a larger part than GRK1b or GRK7b in dark inactivation of the PP1 photoproduct.

GRK7a has been characterized as a cone opsin kinase [29, 30, 33, 34]. To understand whether GRK-involved inactivation of the active photoproduct of bistable PP1 is similar to or largely different from that of UV-sensitive bleaching opsins, we investigated the dark recovery kinetics of the responses in mutant larval PP1 cells in which the opsin SWS1 (which is a cone UV opsin) was expressed instead of PP1. We observed effects similar to those of GRK7a knockdown. Knockdown of GRK7a resulted in a larger peak amplitude, longer peak duration, and remarkably slower recovery of the calcium level in the PP1 cells compared with control, GRK1b-knockdown, and GRK7b-knockdown larvae (Fig. 2e-g, Fig. S4c, d), suggesting that the inactivation profiles of the PP1 and SWS1 opsin photoproducts by GRK7a are similar.

**Fig. 3:**
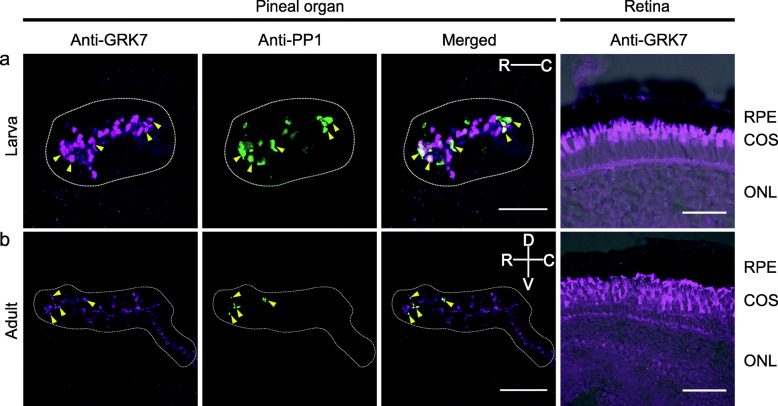
Immunoreactivity of the anti-GRK7 antibody in the PP1 cells of zebrafish larvae and adults. Double immunostaining for GRK7 (magenta) and PP1 (green) in the pineal organ and retina of larval and adult zebrafish using anti-PP1 and anti-GRK7 antibodies. **a** Zebrafish larva (5 dpf, whole-mount preparations); scale bars represent 20 μm. The rostral (R) and caudal (C) sides are shown. **b** Adult zebrafish (tissue sections); scale bars represent 50 μm. The merged image demonstrates that GRK7 is colocalized with PP1 (yellow arrowheads). The white dotted traces indicate the landmarks of the pineal organ. The rostral (R), caudal (C), dorsal (D), and ventral (V) sides are shown

**Fig. 4:**
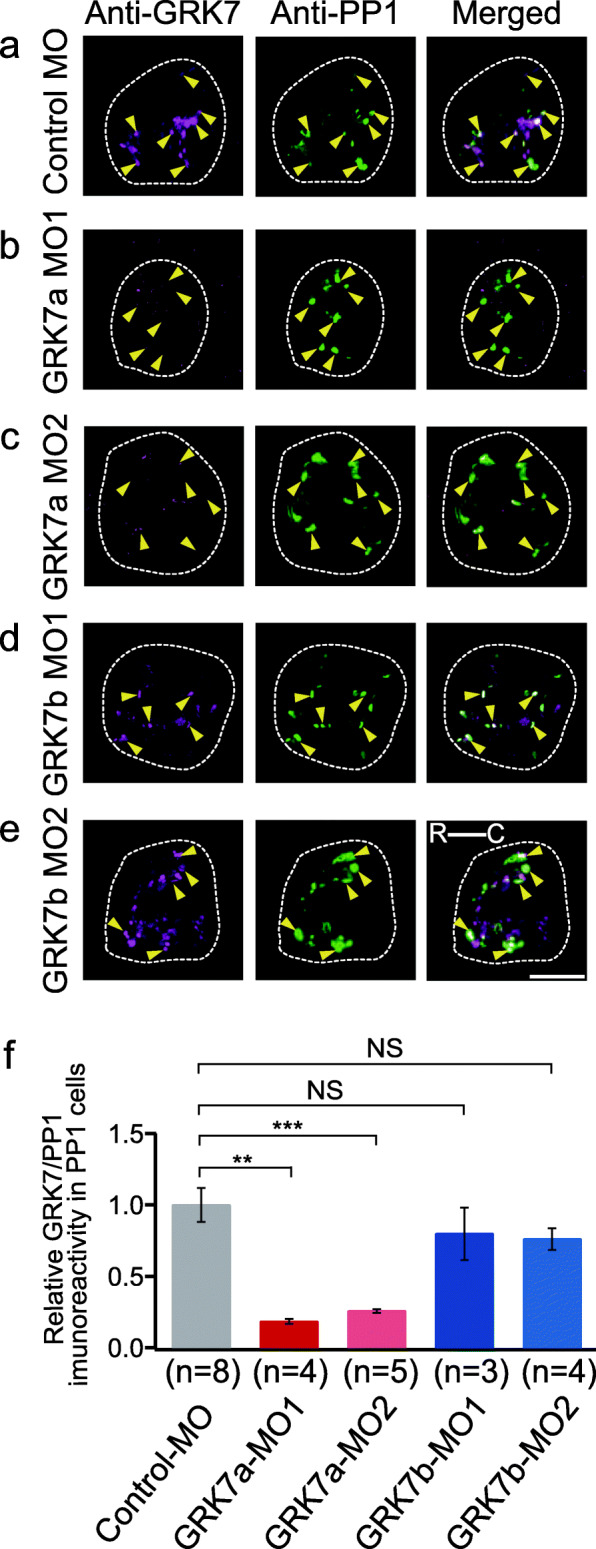
Evaluation of the knockdown effect by immunohistochemistry using an anti-GRK7 antibody. Double immunostaining for GRK7 (magenta) and PP1 (green) in the knockdown zebrafish larvae. **a** Control MO knockdown, (**b**) GRK7a-MO1 knockdown, (**c**) GRK7a-MO2 knockdown, (**d**) GRK7b-MO1 knockdown and (**e**) GRK7b-MO2 knockdown. The white dotted traces indicate the landmarks of the pineal organ. The yellow arrowheads indicate PP1 cells in the pineal organ of larval zebrafish. The scale bar represents 25 μm. The rostral (R) and caudal (C) sides are shown. **f** Quantitative analyses of GRK7 immunoreactivity in different GRK MO- or control MO-injected larvae. The ratios of GRK7 immunoreactivity to PP1 immunoreactivity in PP1 cells are compared. The error bars indicate the standard error. Significance scores represent *P* values determined by Wilcoxon rank sum test (**, *P* < 0.01, ***, *P* < 0.001, NS, nonsignificant, *P* > 0.05)

Finally, we investigated the immunohistochemical distribution of GRK7 in the knockdown larvae shown in Fig. 2 to confirm the expression of GRK7a and GRK7a knockdown by MO-GRK7 in PP1 cells. We performed immunohistochemistry using an anti-carp GRK7 antibody [35] thought to react to both zebrafish GRK7a and GRK7b due to the sequence similarity between the epitope of the antibody in carp GRK7 and corresponding regions of zebrafish GRK7a and GRK7b [29]. We first confirmed that the antibody stained the pineal organs and cones in the retinas of both adult and larval zebrafish (Fig. 3). We investigated the suppression of GRK7a expression in PP1 cells from the knockdown larvae (Fig. 4). Compared with the control MO-injected larvae, the GRK7a-knockdown larvae treated with MO1 or MO2 did not show a clear increase in immunoreaction with the anti-GRK7 antibody (Fig. 4a-c). Quantitative analyses supported this observation (Fig. 4f). On the other hand, immunoreactivity remained higher in the PP1 cells of GRK7b-knockdown larvae treated with MO1 or MO2 (Fig. 4d-f) than in control MO-injected larvae (Fig. 4a). The immunohistochemical results supported our conclusion that GRK7a functionally dominates inactivation of the PP1 photoproduct. To the best of our knowledge, this is the first report to functionally identify the coupling of a GRK to a pineal opsin.

A previous comparison of the initial rates of rhodopsin phosphorylation by recombinant GRKs in zebrafish revealed that rhodopsin phosphorylation activity of GRK7a (GRK7–1) is more than 30-fold faster than those of GRK1a, GRK1b, and GRK7b (GRK7–2 [29]). In carp, GRK7 has much greater phosphorylation activity than GRK1 [35]. Therefore, GRK7a might largely contribute to dark inactivation of the PP1 photoproduct even if GRK1a and/or GRK1b are distributed in PP1 cells.

Because our previous study revealed that PP1 colocalizes with and activates the cone-type transducin Gt2 in zebrafish [16], it can be concluded that upon light absorption, PP1 interacts with a cone-type signal transduction molecule, Gt2, and a cone-type inactivator, mainly GRK7a. A previous study revealed that melanopsin, a nonvisual opsin, was coexpressed with GRK2 and GRK3 in the intrinsically photosensitive retinal ganglion cells of mice [36]. Therefore, each nonvisual opsin might be coupled to different GRKs depending on its molecular properties and function.

In rods and cones, the inactivation of most active opsin photoproducts is achieved via their phosphorylation by an opsin kinase, followed by the binding of arrestin to the phosphorylated photoproduct [18–28]. In addition, the binding of arrestin to the unphosphorylated photoproduct of cone opsins has been reported [37]. Therefore, it is important to investigate how and which arrestin is involved together with GRK7a in the dark inactivation of PP1 photoproducts in the zebrafish pineal organ to understand the dark inactivation mechanism of PP1 in detail. We previously reported that in the lamprey pineal organ, parapinopsin colocalizes with β-arrestin and not visual arrestin [12]. It is well known that β-arrestin binds active G protein-coupled receptors (GPCRs) and facilitates their internalization [38, 39], decreasing the amount of active GPCRs that can activate G protein. Similar to light-insensitive GPCRs, in the lamprey pineal organ, the active parapinopsin photoproduct is internalized after β-arrestin binding and removed from the photoreceptor membranes to avoid activating the G protein [12]. Unlike the color discrimination system in the zebrafish pineal organ, it has been suggested that the lamprey pineal organ employs two types of photoreceptor cells for color discrimination between UV and visible light, i.e., parapinopsin-expressing photoreceptor cells and visible light-sensitive photoreceptor cells [11]. To generate color opponency based on PP1 alone in the zebrafish pineal organ, the amount of active PP1 photoproduct that activates Gt2 has to be almost constant under stable light conditions to maintain the membrane potential level. Accordingly, it can be speculated that PP1 may employ an arrestin that does not mediate internalization, such as visual arrestins, and not β-arrestin. In future studies, we should investigate which types of arrestins bind the PP1 photoproduct to understand the detailed mechanism of dark inactivation of the active PP1 photoproduct.

## Conclusions

In this report, we investigated the effects of GRK knockdown on light responses in PP1 cells in the pineal organs of zebrafish larvae. This was done via calcium imaging with a multiphoton microscope to identify GRKs or opsin kinases that are involved in inactivation of the active photoproduct of PP1. We found that knockdown of a cone opsin kinase, GRK7a, resulted in a larger amplitude, longer time to peak, and prolonged response in PP1 cells compared with those observed in other opsin kinase-knockdown larvae, indicating that the cone opsin kinase GRK7a mainly contributed to this inactivation. We confirmed the expression of GRK7a in the PP1 cells of the larvae and observed similar knockdown effects on the response profile in the PP1 cells of mutant larvae in which a cone opsin, SWS1, was expressed instead of PP1. Taken together, these results suggest that the active photoproduct of PP1 is inactivated by GRK7a in a way similar to the GRK7a-mediated inactivation of cone opsins, which might enable PP1 to generate color opponency without cooperation from other opsins in pineal PP1 cells.

## Materials and methods

### Animals

Zebrafish (*Danio rerio*) were obtained from the Zebrafish International Resource Center and National BioResource Project Zebrafish. The zebrafish were maintained on a 14 h light/10 h dark cycle at 28.5 °C. Embryos (0–7 days postfertilization) were maintained in E3 medium (5 mM NaCl, 0.17 mM KCl, 0.33 mM CaCl_2_, and 0.33 mM MgSO_4_).

### In situ hybridization

The preparation of RNA probes and in situ hybridization were carried out as described previously [16, 40, 41]. Digoxigenin (DIG)- and fluorescein-labeled antisense and sense RNA probes for zebrafish PP1 (full-length coding sequence, accession number, AB626966) and GRK1b (bases 87 to 1087 of the coding sequence, accession number, AY900003), GRK7a (bases 60 to 1060 of the coding sequence, accession number, AY900004), and GRK7b (bases 60 to 1059 of the coding sequence, accession number, AB212996) mRNAs were synthesized using a DIG RNA labeling kit or fluorescein RNA labeling kit (Roche). Sections were pretreated with proteinase K and hybridized with each RNA probe in ULTRAhyb Ultrasensitive Hybridization Buffer (Ambion). For single staining, the probe was detected using an alkaline phosphatase-conjugated anti-digoxigenin antibody (Roche), followed by reaction with 5-bromo-4-chloro-3-indolyl phosphate/nitro blue tetrazolium to stain the sections blue. For double fluorescence labeling, sections hybridized with DIG-labeled probes were incubated with a horseradish peroxidase (HRP)-conjugated anti-DIG antibody (Roche) and subsequently treated with buffer from the TSA plus DNP (HRP) system (Perkin Elmer), followed by incubation with an Alexa 488-conjugated anti-DNP antibody. Fluorescein-labeled probes on the sections were detected by incubation with an alkaline phosphatase-conjugated anti-fluorescein antibody (Roche), followed by a color reaction using the HNPP fluorescent detection set (Roche).

### Morpholino oligonucleotide-mediated knockdown

The sequences of antisense morpholino oligonucleotides (MOs) for zebrafish GRK1b, GRK7a, GRK7b, and the standard control were as follows: GRK1b MO1, 5′–AGTCTCCAAACCTCCAATATCCATG–3′; GRK1b MO2, 5′–CATGTCTTTGTTTTTGTAACTCTCC–3′; GRK7a MO1, 5′–ATCGAGTCCCCCCATGTCACACATT–3′ [30]; GRK7a MO2, 5′–ATTTTGGCAGCTGTGTTGGGTTACC–3′; GRK7b MO1, 5′–CTTCAGTCCACCCATGTCACTCATG–3′; GRK7b MO2, 5′–CATGGCTGTGGCGAGGTGAGGGATC–3′ and standard control MO, 5′–CCTCTTACCTCATTACAATTTATA–3′. Each MO (0.2 mM, ~ 2 nl) was injected into the yolk close to the animal pole in embryos at the one-cell stage obtained from two strains corresponding to the wild-type and SWS1-replacing zebrafish shown in Fig. 2, *Tg(pp1:GCaMP6s)* and PP1^−/−^-based *Tg(pp1:Sws1-P2A-mCherry, pp1:GCaMP6s)*, respectively.

### Immunohistochemistry in tissue sections

Pineal organs and retinas were dissected from adult zebrafish, immersion-fixed in 4% paraformaldehyde (PFA), cryoprotected in 0.1 M phosphate buffer containing 30% sucrose, frozen with OCT compound (Sakura, Tokyo), and sectioned at a thickness of 12–20 μm. The sections were incubated with 1:500-diluted primary antibodies against GRK7 [35] and PP1 [13] in phosphate-buffered saline (PBS) containing 0.3% Triton X-100 and 10% normal goat serum at 4 °C overnight, washed with PBS containing 0.3% Triton X-100 (PBST), and incubated with 1:500-diluted secondary antibodies (Alexa 594- and Alexa 488-conjugated goat anti-rabbit and anti-mouse IgG antibodies, Thermo Fisher Scientific) in PBST at room temperature for 5 h. The sections were sealed with a cover slip using Dako Fluorescent Mounting Medium (Dako North America). Fluorescence images were acquired using a confocal microscope (Leica TCS SP8).

### Whole-mount immunohistochemistry

Whole-mount immunohistochemistry was performed according to the protocol provided in a previous report [42] with slight modifications. In brief, 5-dpf larvae were fixed with 4% PFA overnight at 4 °C, washed with PBST, and treated with 150 mM Tris-HCl (pH 9.0) for 15 min at 70 °C. Subsequently, the larvae were washed with PBST, treated with 0.05% trypsin-EDTA for 45 min on ice, washed in PBST, blocked in PBST containing 1% bovine serum albumin, 2% normal goat serum, and 1% dimethyl sulfoxide (blocking buffer), and incubated in 1:500-diluted primary antibodies in blocking buffer at 4 °C overnight. Note that the GRK7 antibody used to check the expression of GRKs in MO-injected larvae was used at a 1:4500 dilution. Subsequently, the larvae were washed with PBST and incubated with 1:500-diluted secondary antibodies (Alexa 594- and Alexa 647-conjugated goat anti-rabbit and anti-mouse IgG antibodies, Thermo Fisher Scientific) in PBST containing 1% BSA and 1% DMSO. Fluorescence images were acquired using a confocal microscope (Leica TCS SP8). Quantitative analyses of GRK7 immunoreactivity were performed using ImageJ (https://imagej.nih.gov/ij/).

### Two-photon imaging

All conditions used for the current two-photon imaging were in accordance with those described in our previous report [14]. In brief, 5-dpf larvae were mounted dorsally in a low-melting agarose gel (1.5% in E3 medium) on 35 mm glass-bottom dishes (Iwaki). To prevent drying and immobilize the larvae, E3 medium containing 0.002% tricaine (MS222, Sigma) was added to the dishes. Two-photon imaging was performed using an FVMPE-RS instrument (Olympus). The Mai Tai HP DeepSee IR laser (Spectra-Physics) was used for two-photon excitation of GCaMP6s in PP cells. The intensity of the IR laser (930 nm) was 4% of 1.49 W. The fluorescence intensities were calculated using ImageJ (https://imagej.nih.gov/ij/). The mean fluorescence intensity of each region of interest containing PP cells was subtracted from that of a nonfluorescent region outside PP cells (background) to calculate the net fluorescence intensity. To prevent the activation of opsins by two-photon excitation, two-image acquisitions performed before and after the 405-nm laser flash were repeated. Fourteen time intervals (0.5, 1, 2, 3, 5, 7, 9, 10, 15, 20, 25, 30, 35, and 40 s) from the 405 nm laser flash were used for image acquisition. The series of ΔF/F_0_ values for each interval was calculated and plotted against time and are shown as the calcium response profile. The 405 nm laser intensity was ~ 6.0 × 10^14^ photons/cm^2^/s, which was calculated based on the size of the scanning area (~ 0.0064 mm^2^).

## Supplementary Information

**Additional file 1: Supplementary Figure S1.** In situ hybridization of opsin kinases in the zebrafish pineal organ. In situ hybridization showing the mRNA expression of different opsin kinases (GRK1b, GRK7a, and GRK7b) in the pineal organs of adult zebrafish. The rostral (R), caudal (C), dorsal (D), and ventral (V) sides are shown. The scale bar represents 50 μm.

**Additional file 2: Supplementary Figure S2.** Trial of double-fluorescence in situ hybridization in the zebrafish pineal organ using antisense probes for opsin kinases and PP1. Remarkably strong PP1, but not GRK, signals were observed in the zebrafish pineal organs. (a) GRK1b, (b) GRK7a, and (c) GRK7b. The rostral (R), caudal (C), dorsal (D), and ventral (V) sides are shown. The scale bar represents 50 μm.

**Additional file 3: Supplementary Figure S3.** Morpholino oligo (MO)-mediated knockdown of different GRKs in zebrafish larvae**.** (a) Images showing whole bodies of 5 dpf larvae injected with control or different GRK MO2s. Scale bar represents 1 mm. (b) Dorsal view of the pineal organ of 5 dpf larvae injected with control or different GRK MO2s. Landmarks indicate the pineal organ. Scale bar represents 100 μm. (c) Quantitative analyses of pineal size in different GRK MO2- or control MO-injected larvae. The error bars indicate the standard error. Significance scores represent *P* values determined by Wilcoxon rank sum test (NS, nonsignificant, *P* > 0.05).

**Additional file 4: Supplementary Figure S4.** Statistical analysis of peak amplitudes and the initial rate of dark recovery in the light responses between the control and MO-injected zebrafish. The peak amplitudes (a, c) and initial rates of dark recovery (b, d) were statistically analyzed and compared between control and MO-injected wild-type (a, b) and SWS1-replacing mutant zebrafish larvae (c, d). The initial rate of dark recovery was determined by calculating the slope between the peak and the mean point of the next two points after normalizing the calcium change per individual with each peak amplitude. The absolute peak amplitude and initial rate of recovery in both the GRK7a MO1-injected and GRK7a MO2-injected larvae were significantly larger and smaller, respectively, than those of the control larvae in the case of both wild-type and SWS1-replacing mutant zebrafish. However, in the case of the GRK1b MO-injected and GRK7b MO-injected zebrafish, only one or both showed no statistical significance. Significance scores represent *P* values determined by Wilcoxon rank sum test (**, *P* < 0.01, ***, *P* < 0.001, NS, nonsignificant, *P* > 0.05).

## Data Availability

The datasets supporting the conclusions of this article are included within the article.
