# Supplementary figures and images for: Functional identification of an opsin kinase underlying inactivation of the pineal bistable opsin parapinopsin in zebrafish
Source: Zoological Lett. 2021 Feb 12;7:1. doi: 10.1186/s40851-021-00171-1 (PMC7881645; doi:10.1186/s40851-021-00171-1)

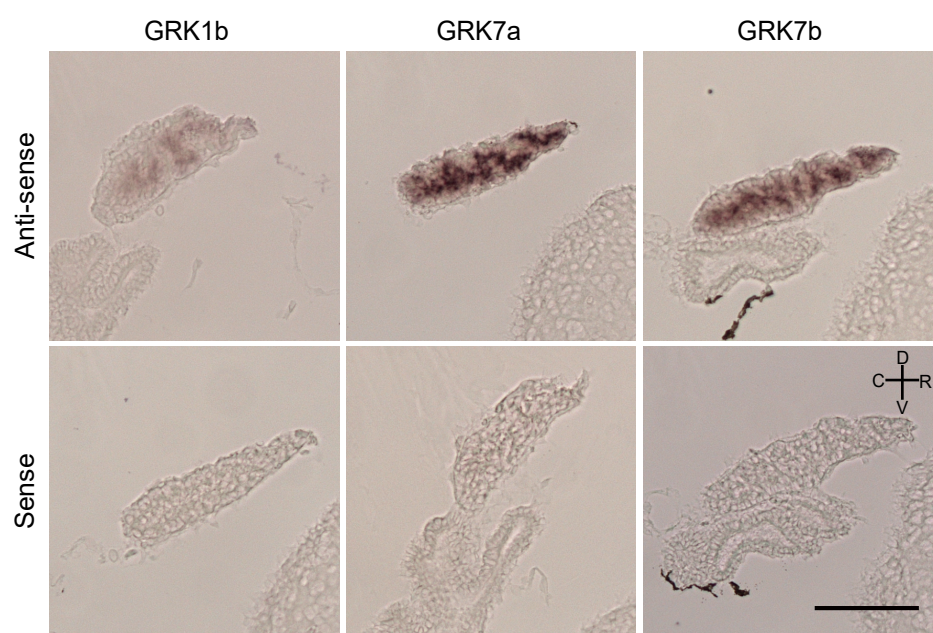

Shen et al, Supplementary Fig. S1

Supplement: Supplementary file 1 — Additional file 1: Supplementary Figure S1. In situ hybridization of opsin kinases in the zebrafish pineal organ. In situ hybridization showing the mRNA expression of different opsin kinases (GRK1b, GRK7a, and GRK7b) in the pineal organs of adult zebrafish. The rostral (R), caudal (C), dorsal (D), and ventral (V) sides are shown. The scale bar represents 50 μm. [file 40851_2021_171_MOESM1_ESM.pdf]

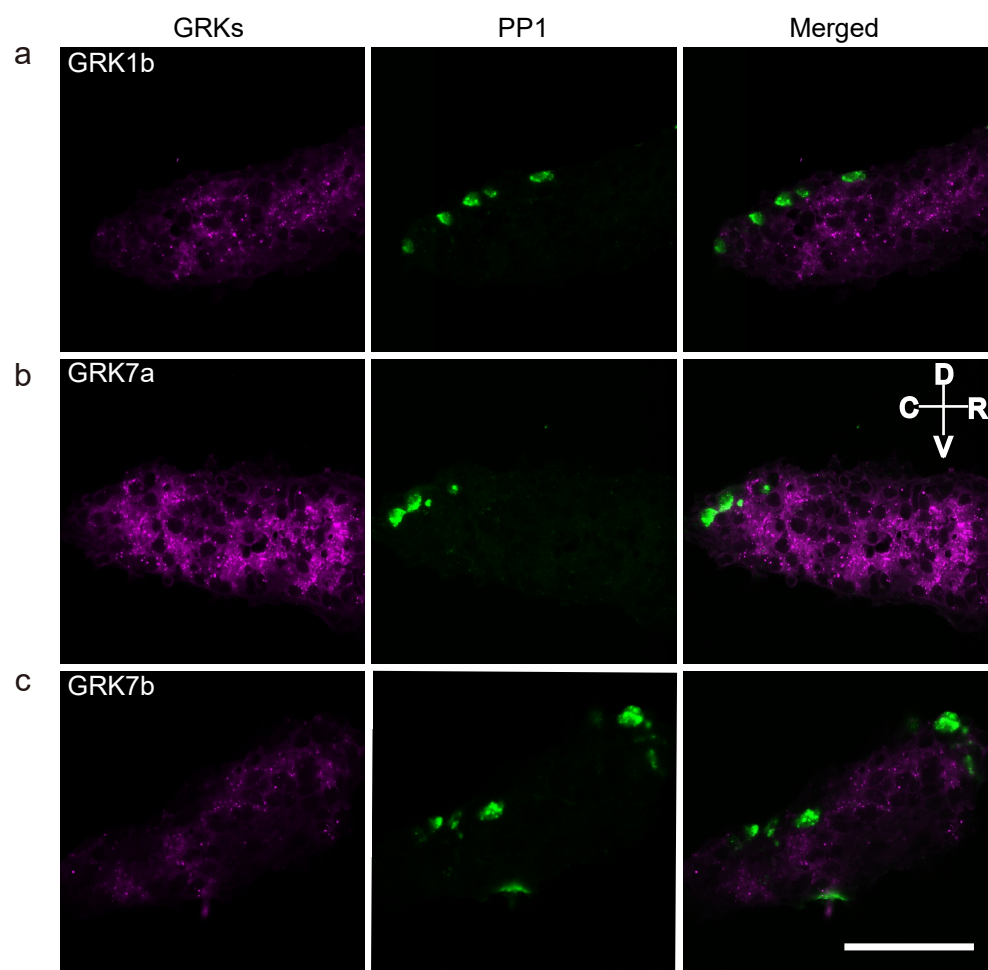

Supplement: Supplementary file 2 — Additional file 2: Supplementary Figure S2. Trial of double-fluorescence in situ hybridization in the zebrafish pineal organ using antisense probes for opsin kinases and PP1. Remarkably strong PP1, but not GRK, signals were observed in the zebrafish pineal organs. (a) GRK1b, (b) GRK7a, and (c) GRK7b. The rostral (R), caudal (C), dorsal (D), and ventral (V) sides are shown. The scale bar represents 50 μm. [file 40851_2021_171_MOESM2_ESM.pdf]

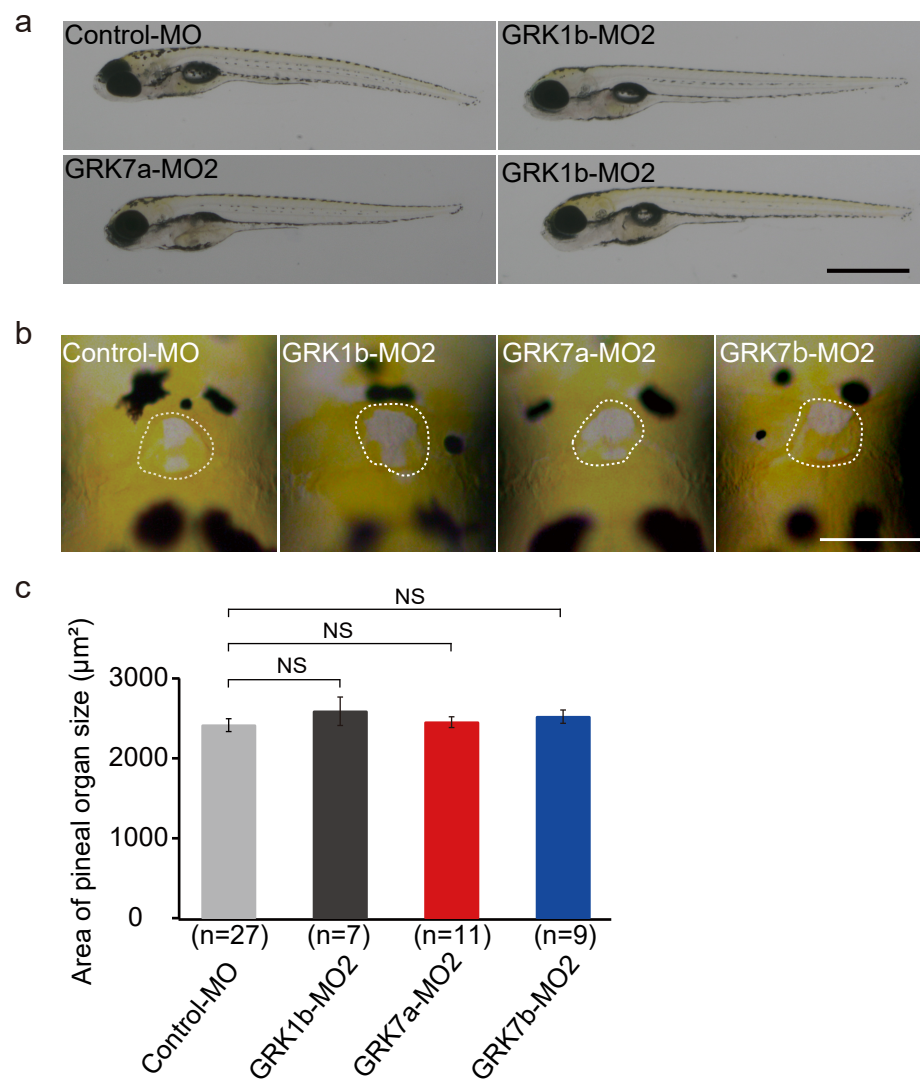

Supplement: Supplementary file 3 — Additional file 3: Supplementary Figure S3. Morpholino oligo (MO)-mediated knockdown of different GRKs in zebrafish larvae. (a) Images showing whole bodies of 5 dpf larvae injected with control or different GRK MO2s. Scale bar represents 1 mm. (b) Dorsal view of the pineal organ of 5 dpf larvae injected with control or different GRK MO2s. Landmarks indicate the pineal organ. Scale bar represents 100 μm. (c) Quantitative analyses of pineal size in different GRK MO2- or control MO-injected larvae. The error bars indicate the standard error. Significance scores represent P values determined by Wilcoxon rank sum test (NS, nonsignificant, P > 0.05). [file 40851_2021_171_MOESM3_ESM.pdf]
